# Posterior cervical necrotising fasciitis: a multidisciplinary endeavour in surgery

**DOI:** 10.1093/jscr/rjad127

**Published:** 2023-03-23

**Authors:** Jia Hui Lee, Fung Joon Foo, Allen Wei-Jiat Wong

**Affiliations:** Department of General Surgery, Sengkang General Hospital, 544886 Singapore; Department of General Surgery, Sengkang General Hospital, 544886 Singapore; Department of Plastic, Reconstructive & Aesthetic Surgery, Sengkang General Hospital, 544886 Singapore

**Keywords:** necrotising fasciitis, abscess, carbuncle, neck, soft tissue infection

## Abstract

A 62-year-old male was diagnosed to the Emergency Department with 5-cm posterior neck carbuncle, and was subsequently discovered to have severe necrotising fasciitis intraoperatively during saucerization of the carbuncle. Subsequently, the patient was admitted to the intensive care unit and underwent combined debridement by the General Surgery, Neurosurgery and Plastic Surgery team. The large defect necessitated a trapezius flap reconstruction for coverage. Three months post-surgery, the patient had recovered well with the full range of movement of his neck.

## INTRODUCTION

The cervical region is susceptible to rapid spread of infection due to proximity to the main vasculature of the head, neck and mediastinum [1]. With a mortality rate of 40–76%, necrotising soft tissue infections (NSTI) of the neck requires early diagnosis, antibiotics and multiple surgical debridement to prevent death [2]. Serial debridement can result in large defects that require reconstruction with soft tissue flaps [3].

## CASE REPORT

A 62-year-old previously healthy male was admitted to the Emergency Department with posterior neck swelling, chills and pain for 3 days. The patient also complained of polydipsia and polyuria.

**Figure 1:**
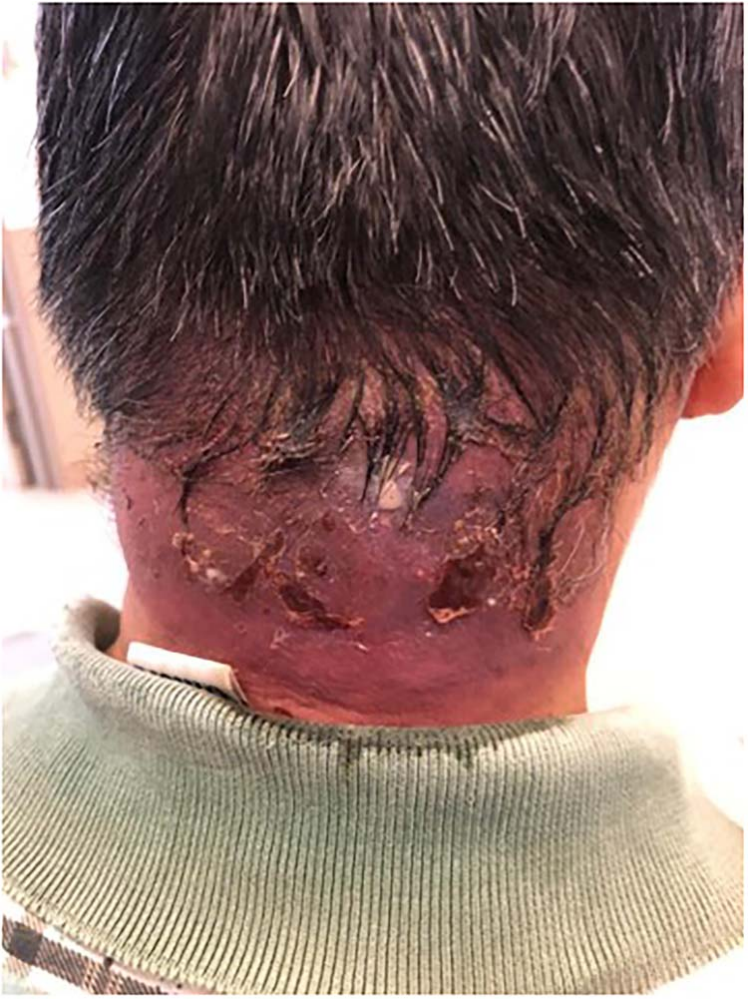
Pre-operative neck carbuncle.

On examination, he had a 5 cm × 2 cm carbuncle over his posterior neck (Fig. 1). His blood glucose level was noted to be 27.3 mmol/L, with serum ketones of 5.2. His White Cell Count was 33 000 and his C-Reactive Protein was 411. He was admitted to the Medical High Dependency for further management of diabetic ketoacidosis, and was commenced on intravenous insulin infusion and amoxicillin-clavulanate acid.

Once the ketosis resolved, he underwent a saucerization of a presumably uncomplicated neck carbuncle by the General Surgery Team.

**Figure 2:**
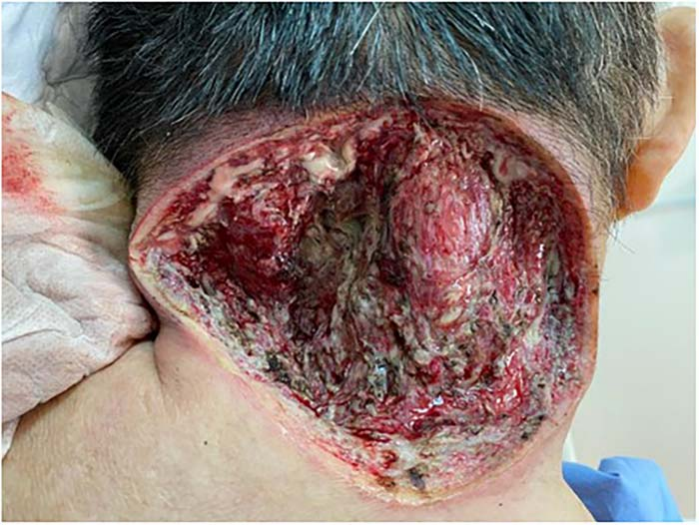
Wound post-saucerization.

Intra-operatively, it was discovered that there was a deep and wide extension of necrotic and purulent tissue to the base of skull (Fig. 2). The debridement was halted once there was close proximity to bone, and an on-table referral was immediately made to the Orthopaedic Team On Call. The decision was made for haemostasis and packing, and for computed tomography (CT) imaging of the neck.

The CT neck showed rim enhancing fluid pockets in the posterior neck muscles bilaterally, and deep to the surgical cavity, without any single dominant collection. The fluid pockets in the suboccipital space were close to the occipital vault outer table, without bony erosion or aggressive periosteal reaction.

After the CT, the patient remained intubated due to a failed air leak test, and was transferred to the intensive care unit (ICU).

His antibiotic regimen was converted to intravenous cefazolin and metronidazole as per Infectious Diseases in view of a Laboratory Risk Indicator for Necrotizing Fasciitis (LIRINEC) score of 8, which suggested a high risk for NSTI [4].

**Figure 3:**
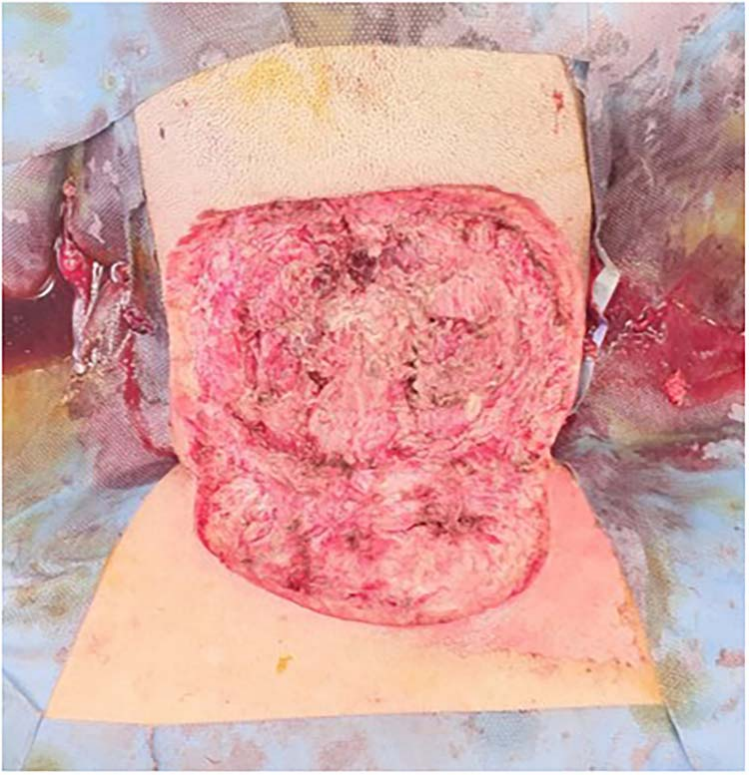
Relook debridement.

With no radiological evidence of bone involvement, the patient underwent a combined debridement by the General Surgery, Neurosurgery and Plastic Surgery unit on day 2 of admission (Fig. 3). Purulent material was discovered to be tracking up to the skull base, inferiorly to C7 and laterally towards the mastoids, in the subcutaneous fat spaces and in-between the muscle belly. There was also noted to be inadvertent debridement of splenius capitis and trapezius during the initial surgery. The occiput, posterior arch of C1, spinous process of C2 and C7 and right lateral mass of C4–5 were palpable but not exposed from coverage by pericranium and periosteum. A washout was performed with iodine, and a negative pressure dressing was applied in view of the 12 cm × 15 cm defect.

Thereafter, the patient underwent trapezius flap reconstruction of his posterior neck wound by the Plastic Surgery unit in view of the large defect, and impending exposure of multiple spinous processes.

### Lower trapezius island fasciomyocutaneous flap based on dorsal scapular artery

**Figure 4:**
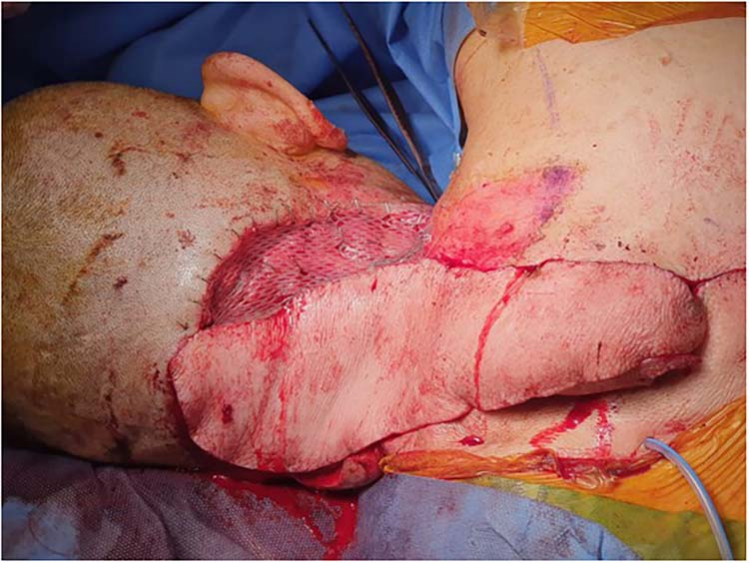
Lower trapezius dorsal scapular artery flap.

**Figure 5:**
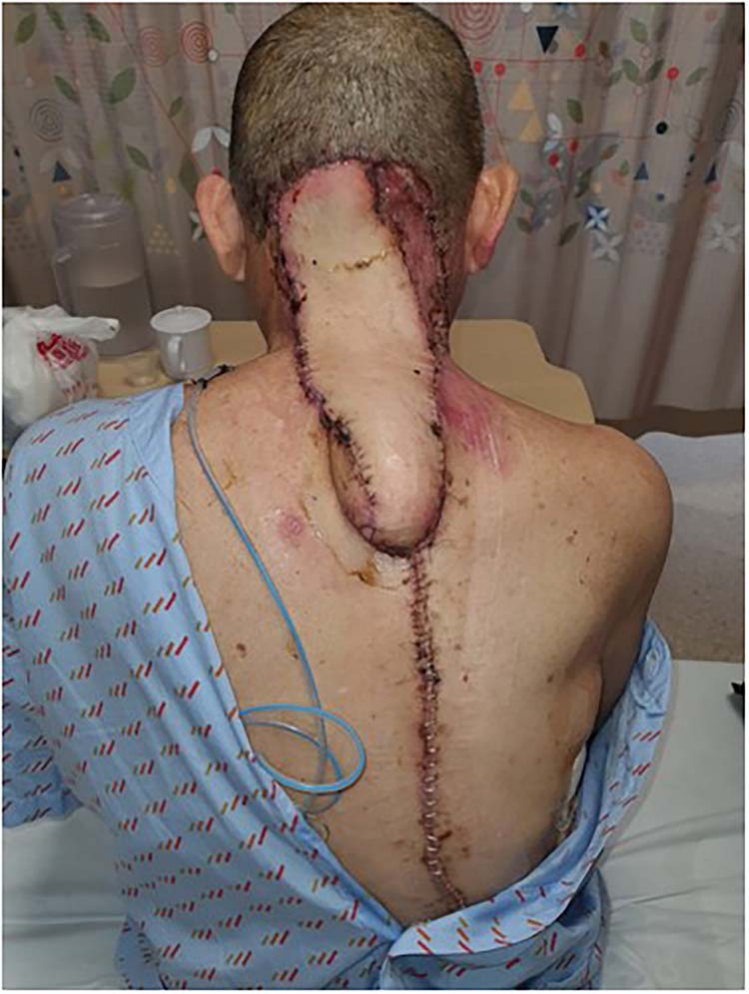
Post-operation day 2.

The trapezius flap was elevated on dorsal scapular artery from inferior to superior, sparing latissimus dorsi, rhomboid major and rhomboid minor. The intervening skin island was divided, flap islanded and transposed into defect, and the remnant right-side defect skin grafted with a split skin graft harvested from the right lateral thigh (Figs 4 and 5). Four drains were inserted to the upper back, upper axillary, lower axillary and neck. The patient was transferred to the High Dependency Unit for further monitoring.

The negative pressure dressing was removed six days after application. The patient underwent a further debridement of superficial epidermolysis of the distal flap with secondary suture 9 days after the trapezius reconstruction. The flap remained healthy, with a small area of dehiscence in the shoulder likely due to patient dependency. The upper back drain was noted to have been dislodged 6 days after insertion, but both axillary drains were removed on day 1, with the final neck drain removed on day 15 post-surgery.

### Outcome and follow-up

The patient remained well, and blood cultures as well as intra-operative wound cultures grew methicillin-sensitive *Staphylococcus aureus* (MSSA). He was discharged after completing 6 weeks of intravenous cefazolin as per the advice of Infectious Diseases. On outpatient review three months post-surgery, the neck wounds were well healed, and there was a full range of movement of the neck (Supplementary Video).

## DISCUSSION

The majority of head and neck NSTI is odontogenic in origin; however, cases of cervical NSTI from trauma, abscess formation and tumour infections have also been reported. Chronic diseases such as diabetes mellitus, hypertension, liver disease and peripheral vascular disease are the main risk factors that predispose patients to developing NSTI [5]. Necrotising fasciitis is often polymicrobial in nature, necessitating administration of broad-spectrum antibiotics, of which the combination of a penicillin and metronidazole is advised [6].

The use of scoring tools such as the LRINEC may aid in diagnosis when there is clinical suspicion, especially in the context of non-specific symptoms [7]. However, despite its high positive and negative predictive values [4], the LRINEC neck tool was found to be of limited usefulness in distinguishing cervical necrotising fasciitis from non-necrotising neck infections [8].

With bone exposure, early flap coverage is important, and a musculocutaneous flap was chosen due to its vascularity and active effect on inhibiting bacterial growth due to oxygen delivery and enhanced inflammatory response [9]. Healing by secondary intention, skin grafts and local flaps were not advised due to the exposed cervical spinous processes and limited tissue vascularity. Regional pedicled flaps can reduce risks compared to free flaps and donor sites [10].

Choi *et al*. described a similar case of NSTI in the posterior neck, with a larger defect about 20–25 cm in size, however without muscle involvement [11]. This was also successfully treated with negative pressure wound therapy and split thickness skin grafting with no restriction of the patient’s range of motion of the neck. Hence, despite the high mortality rate, full recovery from NSTI is possible, if managed early.

## Supplementary Material

Neck_Flap_Movement_rjad127

## Data Availability

The data underlying this article are available in the article and in its online supplementary material.
